# Association of Variability and Pharmacogenomics With Bioequivalence of Gefitinib in Healthy Male Subjects

**DOI:** 10.3389/fphar.2018.00849

**Published:** 2018-08-07

**Authors:** Hong Zhang, Qingmei Li, Xiaoxue Zhu, Min Wu, Cuiyun Li, Xiaojiao Li, Chengjiao Liu, Zhenwei Shen, Yanhua Ding, Shucheng Hua

**Affiliations:** ^1^Phase I Clinical Research Center, The First Hospital of Jilin University, Changchun, China; ^2^The First Affiliated Hospital of Jilin University, Changchun, China

**Keywords:** gefitinib, CYP2D6, pharmacogenetics, pharmacokinetics, food effect

## Abstract

**Objective:** The aim of the study was to explore the association of pharmacokinetic variability and pharmacogenomics with the bioequivalence of orally administered gefitinib (Iressa®, AstraZeneca) provided by three sponsors in healthy subjects.

**Methods:** The study designs were randomized, open-label, and two-period crossover studies in both fasting and fed healthy subjects. In one fasting study, the sample size was enlarged from 30 to 60 for the failing study. Each study subject received a 250-mg gefitinib tablet with a 21-day washout. The plasma concentrations were measured using LC-MS/MS, and pharmacokinetic parameters were determined by noncompartmental methods. Genetic analyses of CYP3A4, CYP3A5, and CYP2D6 alleles were carried out by the polymerase chain reaction (PCR).

**Results:** Two hundred and sixty healthy male subjects were enrolled. The median maximum plasma concentration (T_max_) was 4–5 h, and the mean elimination half-life (t_1/2_) was 18–26 h. The maximum plasma concentration (C_max_) and area under the curve (AUC) increased but T_max_ and t_1/2_ were unaffected by the intake of high-fat food. Three fed and two fasting studies achieved a plausible bioequivalence. The intake of high-fat food decreased the intra-subject variability significantly. In addition, CYP2D6 was associated with gefitinib exposure and may contribute to the high inter-subject variability, but it did not influence the bioequivalence result.

**Conclusions:** Gefitinib is well tolerated, and the bioequivalence is easier to achieve under fed conditions compared to fasting conditions. The 90% confidence interval (CI) of geometric mean ratio (GMR) can be narrowed when the sample size is enlarged without changing the formulation-related technology.

## Introduction

Gefitinib (Iressa®, ZD1839, AstraZeneca) is a highly selective and an orally active small-molecule epidermal growth factor receptor (EGFR) tyrosine kinase inhibitor (TKI). Gefitinib is the first-line treatment used for the patients with metastatic non-small cell lung cancer (NSCLC) (Greenhalgh et al., 2016; Wu and Shih, 2018). Pharmacokinetic studies in humans have shown that gefitinib has a rapid clearance, a high volume of distribution, and an elimination half-life (t_1/2_) of approximately 40 h (Greenhalgh et al., 2016). Although the absolute bioavailability of 250-mg gefitinib tablets in patients has been reported to be approximately 60% but the plasma concentration profiles after oral dosing have shown that the oral administration of gefitinib once a day is suitable, with the steady state being achieved on day 7 (Zhao et al., 2017).

Gefitinib has demonstrated clearance primarily by the hepatic route as the parent compound as well as its metabolites, with less than 4% of the dose being cleared by the renal route (Chen et al., 2018). Furthermore, no apparent relationship has been reported between the occurrence of the CYP3A5 expresser genotype and gefitinib plasma clearance or the t_1/2_. CYP3A4 is the major cytochrome P450 enzyme that is involved in the metabolism of gefitinib, although the formation of the major circulating human metabolite of gefitinib has been shown to be catalyzed primarily by the cytochrome P450 CYP2D6 (Cantarini et al., 2008; Chen et al., 2018). With the end of the patent protection period of the inventor's product, bioequivalence studies have been designed and reported to investigate and compare the pharmacological features of the drug with different formulations pertaining to the extent and rate of absorption of the active ingredient. To this end, in order to corroborate the therapeutic similarity between two drug products incorporating the same active ingredient, bioequivalence data are always crucial. Usually, the determination of bioequivalence depends on comparing the rate and the extent of absorption of the product under study (Test, T) with the original product (Reference, R) (Karalis et al., 2012).

It is a challenge to assess the bioequivalence between the test formulation product (T) and the reference formulation (R) of a drug using a two-way crossover experiment and this challenge has shown great significance and widely considered. In order to claim bioequivalence between two given formulations, the United States Food and Drug Administration (FDA) recommends that the ratio of the two true formulation averages (μ*T*/μ*R*) of pharmacokinetic parameters of concern must lie within some plausible limits (e.g., (80, 120%)), with certain assurance (Karalis et al., 2012).

In any case, the magnitude of the confidence interval (CI) is directly proportional to the intra-subject coefficient of variability (intra-CV) and the difference between the test and reference means, but inversely proportional to the number of participants (N) (Karalis et al., 2012). Therefore, the sample size, intra-CV, and geometric mean ratio (GMR) (T/R ratio) are of significant importance in regard to the fulfillment of bioequivalence criteria (Ramirez et al., 2008; Hirose et al., 2016). Both fasting and fed studies conducted in healthy males are recommended by the FDA and the China Food and Drug Administration (CFDA) for gefitinib bioequivalence study guidance. Our literature survey has stemmed that the effect of high fat food intake and CYP gene polymorphisms on the pharmacokinetics of gefitinib tablets as well as the variability of bioequivalence studies have not been reported. Bioequivalence studies generally emphasize the release of the active moiety from a drug formulation and its subsequent absorption into systemic circulation. In this study, we aimed to compare the bioequivalence of two 250-mg gefitinib tablets obtained from three different sponsors (T) and AstraZeneca Plc (Iressa®, R). We further investigated the effect of high-fat food intake, intra-subject and inter-subject variability, pharmacogenomics, and sample size on the bioequivalence of gefitinib.

## Materials and methods

### Study design

**Figure 1:**
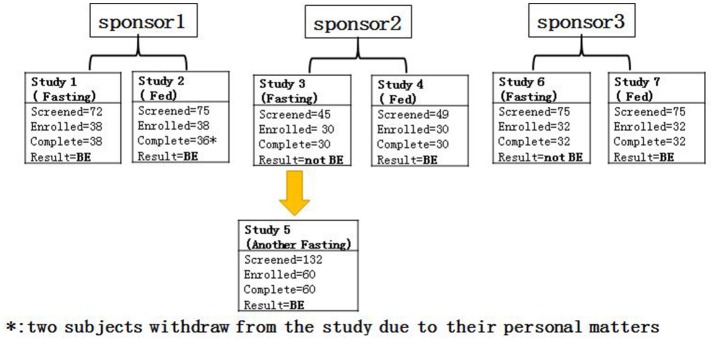
The flow chart of the study.

Seven gefitinib bioequivalence studies were conducted to compare the bioequivalence of two 250-mg gefitinib tablets from three different sponsors (T) and AstraZeneca Plc (Iressa®, R). These study designs were randomized, open-label, and 2-period crossover studies in both fasting and fed healthy male subjects. Study 1 (*n* = 39) and study 2 (*n* = 39) were conducted under fasting and fed conditions, respectively, to determine the bioequivalence of gefitinib from sponsor 1. Meanwhile, study 3 (*n* = 30) and study 4 (*n* = 30) were conducted under fasting and fed conditions, respectively, to determine the bioequivalence of gefitinib from sponsor 2. Study 5 (*n* = 60) was an additional study investigating the bioequivalence of gefitinib from sponsor 2 under different fasting conditions. Finally, study 6 (*n* = 32) and study 7 (*n* = 32) were executed under fasting and fed conditions, respectively, to establish the bioequivalence of gefitinib from sponsor 3 (Figure 1). According to the FDA guidelines^1^, these studies were randomized, open-label, single-dose, two-treatment, two-sequence, and two-period crossover studies to assess the bioequivalence of the R and T formulations of gefitinib. Gefitinib 250-mg tablets were administered under fed/fasting conditions and conducted in the Phase I Clinical Trials Unit at The First Hospital of Jilin University, Changchun, Jilin, China. Each subject was randomized to one of two treatment sequences (TR, RT), according to a randomization schedule prepared prior to the study. There was a 3-week washout period between each single dosing. Subjects were dosed at the same time on day 1 and 22 in all studies (1–7).

### Inclusion and exclusion criteria

Healthy male, Chinese volunteers, aged 18–65 years old, were enrolled after obtaining written informed consent and were eligible if they had no clinically relevant conditions identified from their medical history, physical examination, laboratory investigations, and electrocardiogram. The use of any drug known to induce or inhibit cytochrome P450 or responsible for modifying gastric pH by the study subjects within 4 weeks before dosing was considered as a major exclusion criterion. All study subjects were provided written informed consent to become a volunteer participant in study followed by an interpretation of study procedures. This study was conducted at The First Hospital of Jilin University, Changchun, Jilin, China, in accordance with Good Clinical Practice and the Declaration of Helsinki. In addition, this study was approved by an independent ethics committee at The First Hospital of Jilin University, Changchun, Jilin, China, before the trial commenced.

The eligible subjects were directed to stay in the research center for 48 h prior to drug administration (pre-dosing) and were not allowed to leave the research facility. After 12 h of fasting, each study subject received 240 mL of water for dosing.

For the fed bioequivalence studies, the study subjects consumed high-fat food, which they started to consume approximately 30 min prior to dosing. The subjects were discharged from the research facility at 48 h post-dose. Blood samples were collected from each study subject at the remaining time points. The subjects were continuously monitored by investigators of the current study throughout the study period. Vital signs (blood pressure, pulse, and temperature) were measured at time 0 (within 60 min pre-dose) and at 3, 6, 8, 24, 48, 72, 96, 120, 144, 168, 192, and 216 h post-dose in study 1 and study 2. The time points were 0 (within 60 min pre-dose) and 3, 6, 12, 24, 48, 72, 96, 120, 144, and 168 h post-dose in studies 3–7. The subjects were advised to remain sitting in a semireclined posture, and ambulation was limited within 4 h post-dose. On the day of dosing, a light lunch and dinner were taken at 4 and 10 h after drug administration, respectively. In studies 1–7, at 1 h prior to drug administration and 2 h post-drug administration, the water intake restriction was strictly followed. The consumption of grapefruits, grapefruit juice, liquorice, poppy seeds, oranges, and strenuous physical exercise were not allowed for 72 h prior to the drug dose until 168 h after each drug dose. Similarly, alcohol consumption and intense physical activity were not allowed during the study period of 48 h prior to drug administration until 168 h after each drug dose. Concomitant mediations except for paracetamol were not allowed from 72 h before the first drug dose until completion of the study.

### Estimation of sample size

According to the current FDA guidelines (Karalis et al., 2012), to achieve 80% power (1-β) at the 5% nominal level (α = 5%), the GMR is usually set at 95–105%. The coefficient of variation (CV) is the intra-subject variability (intra-CV); for gefitinib, the intra-CV is reported to be 17–30% (Cantarini et al., 2008), wherein the sample size used initially was 14–38, which was estimated by PASS Version11 software. Based on the above sample size estimation results as well as the opinions of the sponsors and investigator, the sample sizes used in the studies are shown in Table 1. Study 3 did not achieve plausible bioequivalence. To execute study 3, we referred to the GMR and intra-CV for this study (study 3) and redesigned another bioequivalence study (study 5, another fasting study) with the same operating procedure and drug formation except for the different sample size (Table 1).

**Table 1:** The sample size estimation in studies 1–7.

**Sponsor**	**Study number**	**Food effect**	**Predicted bioavailability**	**α**	**1-β**	**Intra-Subject variability**	**Sample size estimation**	**Sample size in the study**
1	1	Fasting	0.95–1.05	0.05	0.8	17–30%	14–38	39
1	2	Fed	0.95–1.05	0.05	0.8	17–30%	14–38	39
2	3	Fasting	0.95–1.05	0.05	0.8	17–30%	14–38	30
2	4	Fed	0.95–1.05	0.05	0.8	17–30%	14–38	30
2	5	Another fasting	1.08	0.05	0.8	30%	53	60
3	6	Fasting	0.95–1.05	0.05	0.8	17–30%	32	32
3	7	Fed	0.95–1.05	0.05	0.8	17–30%	32	32

### Pharmacokinetic analysis

For the gefitinib pharmacokinetic assay, venous blood (5 mL) was drawn into K_2_EDTA anticoagulant tubes at pre-dose and 0.25, 0.5, 1, 2, 3, 4, 5, 6, 7, 8, 10, 12, 24, 36, 48, 72, 96, 120, 144, 168, 192, and 216 h post-dose in studies 1 and 2. The pharmacokinetic time points were pre-dose and at 1, 2, 3, 4, 5, 6, 7, 8, 9, 10, 12, 24, 48, 72, 96, 120, 144, and 168 h post-dose in studies 3–7. Blood samples were centrifuged (3,000 rpm, 10 min) within 30 min, and the plasma was stored at −80°C until analysis. High-performance liquid chromatography coupled with tandem mass spectrometry (HPLC–MS/MS, AB SCIEX Triple Quad 6500, USA) was employed for determination of the gefitinib concentration at WuXi AppTec, China. The chromatographic system consisted of a C_18_ column (ODS, 150 mm × 4.4 mm i.d., 5μm), and the *m/z* **–**447/128 (precursor/product) was monitored for gefitinib. The lower limit of quantification (LLOQ) was 0.5 ng/mL, and the validated concentration range was 0.5–500 ng/mL.

### Genetic analysis

The investigation revealed that the plasma concentration varied greatly, and varying bioequivalence results were observed in studies 3 and 5. It was envisioned to further explore the relationship between the outcome and CYP genetic polymorphisms. To this end, pre-dose blood samples (4 mL) for CYP3A4, CYP3A5, and CYP2D6 genotype assays were collected in K_2_EDTA anticoagulation tubes and stored at −80°C until polymerase chain reaction (PCR) analysis. The CYP genotype assay was performed using a real-time PCR instrument (Bio-Rad Laboratories, Hercules, CA, USA) at Shanghai Bohao Biotechnology Co., Ltd.

### Clinical description of study subjects

In this study, for each subject, the medical history, physical examination, electrocardiogram, and laboratory tests such as hematology, biochemistry, and urinalysis, etc. were performed at screening (2–14 days before the first drug dose of the study) and also at the end of the study. Vital signs (blood pressure, pulse, and temperature) were recorded immediately before the first dose and post-dosing. Adverse events (AEs) were recorded daily from the day of the first dose until the end of the study.

### Pharmacokinetic and statistical analyses

Pharmacokinetic analysis was performed using WinNonlin Professional, Version 6.4, software (Pharsight Corporation, Cary, NC, USA), and the plasma concentration vs. time data (pharmacokinetic parameters) were analyzed by using a noncompartmental method. The pharmacokinetic parameters for gefitinib included the maximum plasma concentration (C_max_), area under the concentration–time curve from 0 h to the last time point (AUC_0−t_), area under the concentration–time curve from 0 h to infinity (AUC_0−∞_), time of maximum plasma concentration (T_max_), and t_1/2._ Descriptive statistics were calculated for the pharmacokinetic parameters; however, the demographic and safety variables were analyzed by the *T*-test or analysis of variance (ANOVA) model. CYP genotypes were compared by the chi-squared test. ANOVA was used to compare the AUC and C_max_ using the factors fitted for the effect of sequence, subject within sequence, period, and treatment. The comparison was presented in terms of the geometric least-square means, and the 90% CI. Bioequivalence was declared if the 90% CI of the treatment ratio was within the equivalence range of 0.8–1.25. T_max_ and t_1/2_ were analyzed with the Wilcoxon signed-rank test. All statistical tests were performed by SAS 9.1 Statistical Package. *P* < 0.05 was considered statistically significant.

## Results

### Subject screening, recruitment, and compliance

A total of 523 healthy male subjects were initially screened for these studies. Of these, 262 subjects met the study criteria and enrolled in the study, and 260 subjects were in compliance with the study protocols. Most of the study subjects were Han Chinese. The mean age of the each study subjects was 28.9–39.4 years old and the mean age of the total subjects was 34.1±9.0 years old. However, the study subjects were older in studies 6–7, compared with those enrolled in studies 1–5. The mean body mass index (BMI) was 23.1 ± 2.4 kg/m^2^, and there was no significant BMI difference between each study. The demographics of the enrolled subjects are shown in Table 2. All the subjects were included in the safety analysis set, and 258 subjects were included in the pharmacokinetic analysis set (Table 2, Figure 1).

**Table 2:** Demographic characteristics of the healthy male volunteers^*^.

**Sponsor**	**Study number**	***N***	**Age [years, mean (SD)]**	**Ethnicity (Han/Other)**	**Body mass index [kg/m^2^, mean (SD)]**
1	1	39	31.9 (8.2)	35/3	22.7 (2.2)
1	2	39	31.6 (7.2)	35/3	22.8 (2.0)
2	3	30	28.9 (7.4)	29/1	23.1 (2.3)
2	4	30	33.4 (5.8)	28/2	23.5 (2.6)
2	5	60	32 (8.3)	56/4	23 (2.4)
3	6	32	38.6 (10.8)#	32/0	23.7 (2.8)
3	7	32	39.4 (10.6)#	32/0	22.9 (2.4)

* *All subjects were male; # P < 0.05 compared with age in studies **1–5**; there was no significant difference between studies **6** and **7***.

### Gefitinib plasma concentration–time profiles

**Figure 2:**
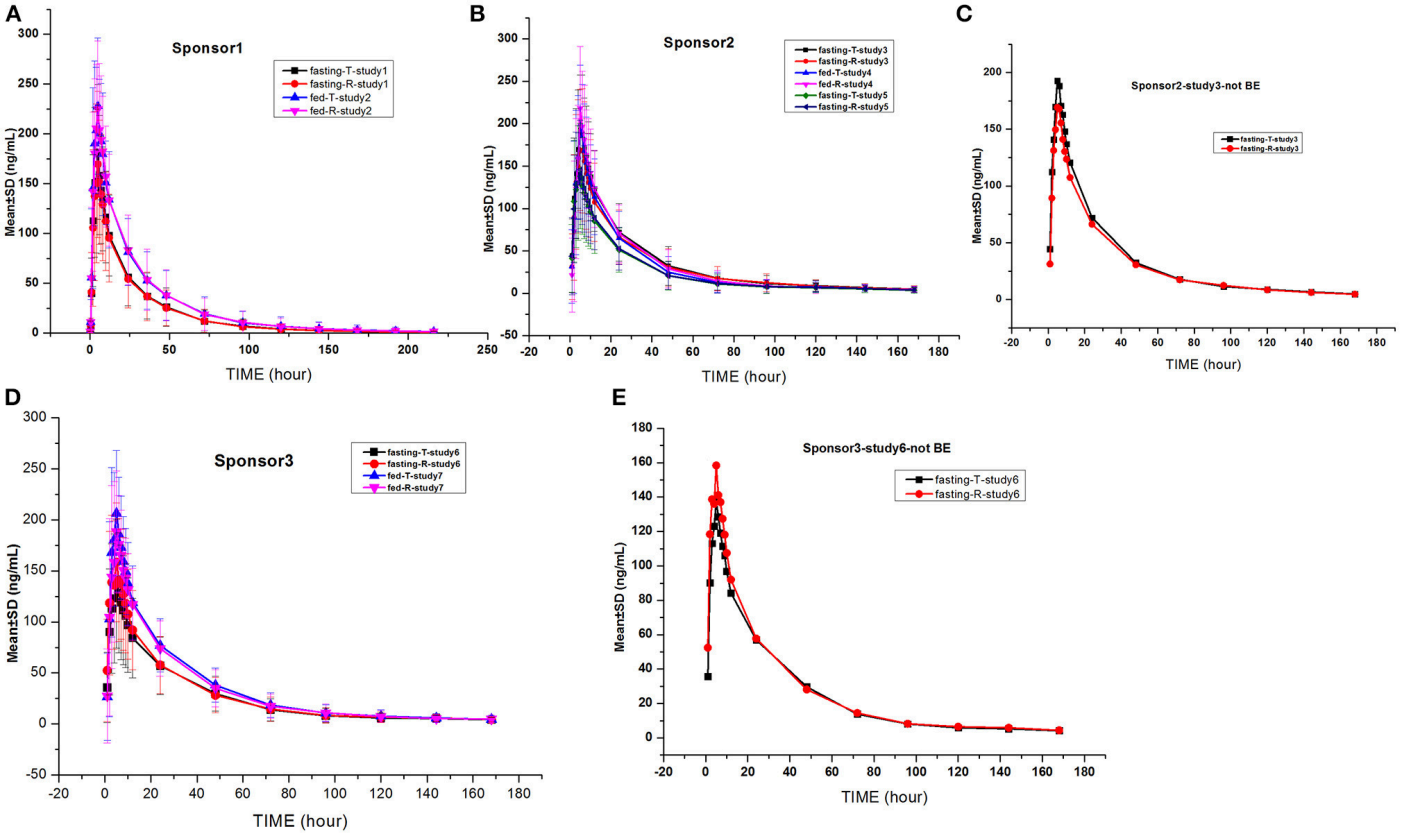
Mean gefitinib plasma concentration vs. time profiles in the studies: The drug was provided by sponsor 1 **(A)**, sponsor 2 **(B,C)**, and sponsor 3 **(D,E)**. Bioequivalence was not observed in the studies shown in **(C,E)**.

**Figure 3:**
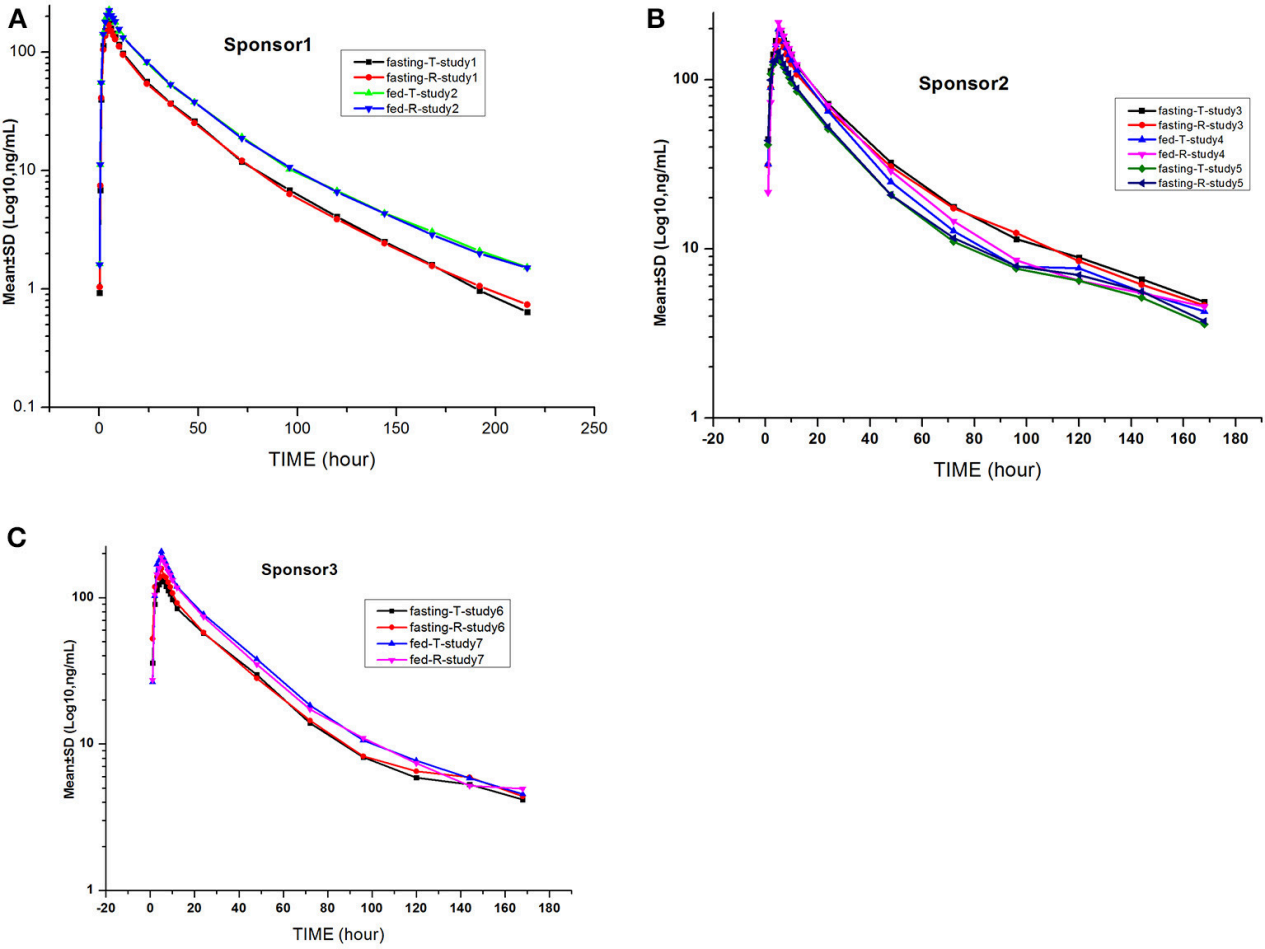
Mean log gefitinib plasma concentration vs. time profiles in the studies: The drug was provided by sponsor 1 **(A)**, sponsor 2 **(B)**, and sponsor 3 **(C)**.

The gefitinib concentrations of plasma samples which exhibited lower than the LLOQ (0.5 ng/mL) were recorded as zero before the C_max_ and were not detectable after the peak. The gefitinib plasma concentration vs. time profiles are illustrated in Figures 2, 3. The gefitinib plasma concentrations increased slowly in all study subjects and reached a median C_max_ of 141–248 ng/mL at 4–5 h post-dose. The plasma concentrations showed a decline in a biphasic manner, which initially decreased rapidly and then demonstrated a slightly decrease until the lower limit of quantification, and the geometric mean of t_1/2_ was reached at 18–26 h. The mean plasma concentration vs time profile of gefitinib over time was similar in the R and T formulations in studies 1, 2, 4, 5, and 7. However, the observed gefitinib plasma concentrations vs. time profile of the T formulation was higher compared to the R formulation in study 3. Furthermore, the gefitinib plasma concentration vs. time profile of the R formulation was higher compared to the T formulation in study 6.

### Pharmacokinetic parameters of gefitinib in studies 1–7

As described previously, the pharmacokinetic parameters were estimated using a non-compartmental model. Table 3 represents the main pharmacokinetic parameters of gefitinib such as AUC_0−t_, AUC_0−∞_, C_max_, T_max_, and t_1/2_. AUC_0−t_ accounted for more than 90% of the total AUC_0−∞_ in all of the study subjects, indicating that the plasma concentration vs. time profiles were well characterized. The CV values of the pharmacokinetic parameters for the R formulation and the T formulation were similar but very large, and they were almost more than 50% (Table 3).

**Table 3:** The pharmacokinetic parameters of gefitinib in each study (Geometric Mean (CV%)).

**Sponsor**	**Study**	**Food**	**Med**	**AUC_0−∞_ (h·ng/mL)**	**AUC_0-t_ (h·ng/mL)**	**CL (mL/h)**	**t_1/2_ (h)**	**V (mL)**	**C_max_ (ng/mL)**	**T_max_ (h)**
1	1	Fasting	R	3,516 (74)	3,475 (74)	71,103 (74)	21 (68)	2,161,393 (48)	175 (13–444)	5 (2–24)
1	1	Fasting	T	3,659 (72)	3,627 (72)	68,327 (72)	19 (61)	1,914,768 (36)	200 (37–317)	5 (3–12)
1	2	Fed	R	5,559 (54)	5,483 (53)	44,971 (54)	25 (65)	1,634,600 (42)	246 (108–399)	5 (1–10)
1	2	Fed	T	5,536 (54)	4,992 (84)	45,156 (54)	26 (60)	1,697,296 (47)	248 (108–471)	4 (2–7)
2	3	Fasting	R	4,381 (58)	4,268 (57)	57,067 (58)	22 (60)	1,808,606 (43)	183 (76–370)	5 (2–24)
2	3	Fasting	T	4,673 (67)	4,557 (66)	53,499 (67)	21 (63)	1,655,473 (46)	212 (57–382)	5 (2–7)
2	4	Fed	R	4,460 (56)	4,385 (55)	56,055 (56)	19 (52)	1,502,119 (32)	223 (117–403)	5 (2–7)
2	4	Fed	T	4,115 (57)	4,039 (55)	60,755 (57)	18 (55)	1,621,255 (28)	211 (109–374)	5 (2–8)
2	5	Another fasting	R	3,315 (62)	3,232 (61)	75,424 (62)	18 (59)	2,006,300 (48)	162 (27–303)	5 (1–24)
2	5	Another fasting	T	3,239 (56)	3,167 (56)	77,192 (56)	18 (61)	2,031,828 (52)	160 (34–288)	5 (2–24)
3	6	Fasting	R	3,914 (56)	3,823 (55)	63,867 (56)	22 (49)	2,013,928 (38)	162 (68–308)	5 (2–8)
3	6	Fasting	T	3,570 (69)	3,461 (70)	70,034 (69)	22 (51)	2,219,308 (51)	141 (38–272)	5 (2–24)
3	7	Fed	R	5,046 (46)	4,909 (45)	49,548 (46)	24 (56)	1,732,107 (44)	198 (108–383)	5 (2–12)
3	7	Fed	T	5,351 (44)	5,224 (43)	46,723 (44)	24 (44)	1,628,739 (33)	213 (101–394)	5 (2–7)

No apparent differences were seen in either the absorption or elimination rates of the R or T formulation of gefitinib, as shown by identical values for different pharmacokinetic parameters in studies 1, 2, 4, 5, and 7. However, more absorption and less elimination of the T formulation compared to the R formulation were observed in study 3. On the contrary, opposite results were found in study 6 (Table 3).

It was noticed that the C_max_ and AUC were in the range of 1.26–1.58 and 1.12–1.58, respectively. The clearance and volume showed a decrease and were in the range of 0.63–0.89 and 0.73–0.9, respectively, in the study subjects fed high-fat content food. However, the T_max_ and t_1/2_ remained unaffected. It was inferred that high-fat food intake could possibly increase the absorption of gefitinib (Table 4).

**Table 4:** The ratio and the *P*-values of pharmacokinetic parameters of the fed vs. fasting study with the test drug from the same sponsor.

**Sponsor**	**Drug**	**AUC_0-t_**	**AUC_0−∞_**	**CL**	**t_1/2_ (h)**	**V**	**C_max_**	***p* for T_max_#**
1	R	1.58 (1.26–1.98)	1.58 (1.26–1.98)	0.63 (0.50–0.79)	>0.05	0.76 (0.64–0.89)	1.48 (1.23–1.78)	>0.05
1	T	1.38 (1.06–1.79)	1.51 (1.21–1.90)	0.66 (0.53–0.83)	0.023	0.89 (0.76–1.03)	1.38 (1.18–1.62)	0.003
2	R	1.24 (1.02–1.50)	1.23 (1.01–1.49)	0.82 (0.67–0.99)	>0.05	0.78 (0.67–0.90)	1.41 (1.20–1.65)	>0.05
2	T	1.13 (0.93–1.37)	1.12 (0.92–1.37)	0.89 (0.73–1.08)	>0.05	0.85 (0.73–0.99)	1.34 (1.13–1.58)	>0.05
3	R	1.28 (1.05–1.56	1.29 (1.05–1.58)	0.78 (0.63–0.95)	>0.05	0.86 (0.73–1.01)	1.26 (1.10–1.45)	>0.05
3	T	1.51 (1.21–1.89)	1.50 (1.20–1.87)	0.67 (0.53–0.83)	>0.05	0.73 (0.62–0.87)	1.58 (1.33–1.87)	>0.05
2^*^	R	1.32 (1.07–1.62)	1.32 (1.07–1.63)	0.76 (0.61–0.93)	>0.05	0.90 (0.76–1.06)	1.13 (0.95–1.36)	0.015
2^*^	T	1.44 (1.17–1.77)	1.44 (1.17–1.77)	0.69 (0.56–0.85)	>0.05	0.81 (0.68–0.97)	1.40 (1.17–1.69)	0.038

* *Another fasting (study 3) vs. fasting (study 5) with drug from sponsor 2; # the Wilicoxon test was used to compare the T_max_ between the fasting study and the fed study; e.g., the AUC_0−t_ of R under fed conditions vs. AUC_0−t_ of R under fasting conditions with drug from sponsor 1 from Table 3 is equal to 5483/3475 = 1.58*.

Although the drug formulation was the same, the exposure was higher in study 3 compared to study 5 with drug from sponsor 2. Forty-six single nucleotide polymorphisms (SNPs) in the CYP3A4, CYP3A5, and CYP2D6 regions were examined, and the alleles conformed to the law of Hardy–Weinberg equilibrium. We found that 10 SNPs showed an association with different exposures. However, nine SNPs were not different in studies 3 and 5, and CYP2D6 rs1058164 was significantly associated with high exposure in study 3 (Table 6, Figure 5).

### Bioavailability and bioequivalence analysis

Three fed studies (2, 4, and 7) and two fasting studies (1 and 5) achieved bioequivalence, and two fasting studies (3 and 6) did not achieve bioequivalence. The 90% CIs for the ratio of the logarithmically transformed pharmacokinetic parameters of gefitinib are shown in Table 5. Unfortunately, the ratio of the T vs. R formulation for C_max_ was 1.15, and the 90% CI was 1.02–1.30, which is greater than 1.25 in study 3. The ratio of the T vs. R formulation for C_max_ was 0.84, and the 90% CI was 0.75–0.94, which is less than 0.8. So, studies 3 and 6 also did not satisfy the bioequivalence criteria (Table 5).

**Table 5:** Bioequivalence assessment summary and re-estimation of sample size.

**Study**	**Pharmacokinetic parameter**	**C_max_**	**AUC_0-t_**	**AUC_0−∞_**	**Re-estimated sample size**
Study 1 (Fasting)	GMR (90%CI)	1.08 (0.96–1.22)	1.04 (0.97–1.12)	1.08 (0.96–1.11)	58
	intra-CV	31.9	18.55	18.38	
	inter-CV	48.75	69.68	69.86	
Study 2 (Fed)	GMR (90%CI)	1.02 (0.97–1.08)	0.97 (0.93–1.02)	0.97 (0.93–1.02)	8
	intra-CV	13.11	11	11.1	
	inter-CV	28.23	54.68	55.97	
Study 3 (Fasting)	GMR (90%CI)	1.15 (1.02–1.30)	1.06 (0.97–1.16)	1.06 (0.97–1.16)	NA
	intra-CV	28.06	20.75	20.56	
	inter-CV	35.16	55.13	56.18	
Study 4 (Fed)	GMR (90%CI)	0.95 (0.9–1.01)	0.92 (0.87–0.96)	0.92 (0.88–0.96)	10
	intra-CV	12.54	10.82	10.92	
	inter-CV	27.38	54.59	53.59	
Study 5 (Fasting)	GMR (90%CI)	0.93 (0.85–1.02)	0.97 (0.91–1.04)	0.97 (0.91–1.03)	52
	intra-CV	30.88	21.37	20.96	
	inter-CV	42.7	53.7	54.5	
Study 6 (Fasting)	GMR (90%CI)	0.84 (0.75–0.94)	0.90 (0.81–1.00)	0.91 (0.82–1.00)	NA
	intra-CV	27.35	24.36	22.9	
	inter-CV	36.18	56.73	57.12	
Study 7 (Fed)	GMR (90%CI)	1.05 (0.9–1.12)	1.06 (1.00–1.12)	1.06 (1.00–1.13)	12
	intra-CV	14.53	13.54	13.36	
	inter-CV	26.47	40.3	41.5	

*AUC0-t, area under the concentration–time curve from 0 h to the last time point; Cmax, maximum plasma concentration; AUC0-∞, area under the concentration–time curve from 0 h to infinity; t1/2, terminal half-life; CI, confidence interval; GMR, geometric mean ratio*.

**Table 6:** Association between pharmacogenomic analysis with pharmacokinetic data.

**Study**	**Genotype (CYP2D6 rs1058164)**	**Number (%)**	**R formulation**		**T formulation**	
			**AUC_0-t_ mean (CV%)**	**AUC_0−∞_, mean (CV%)**	**AUC_0-t_ mean (CV%)**	**AUC_0−∞_, mean (CV%)**
3	C/C	3 (10)	8006.18 (44.09)	8548.49 (43.64)	7365.45 (26.23)	7929.72 (27.69)
	C/G	20 (66.67)	5039.97 (46.43)	5174.93 (47.81)	5708.25 (44.36)	5852.33 (45.72)
	G/G	7 (23.33)	3037.05 (37.95)	3082.73 (37.96)	3155.95 (47.66)	3200.83 (47.19)
5	C/C	0 (0)				
	C/G	48 (84.21)	3874.21 (50.58)	3975.65 (52.03)	3785.7 (54.47)	3869.11 (55.44)
	G/G	9 (15.79)	3162.07 (61.63)	3371.06 (68.34)	3090.56 (69.73)	3267.96 (75.24)
p^*^		0.029	0.001	0.001	0.01	0.01

* *The chi-squared test was used to compare genotypes between study 3 and study 5; ANOVA was used to compare the AUC between different genotypes*.

### Inter-subject and intra-subject variability analyses

The inter-CV of AUC was about 50%, which is more than that of C_max_ in each fasting study; and the intra-CV of C_max_ was in the range of 27.35–31.9% in the fasting study. However, the intra-CV values of C_max_ and AUC were similar, even if the intra-CV of C_max_ was relatively higher than that of AUC; and the intra-CV of C_max_ was about 12.54–14.53% in the fed study.

**Figure 4:**
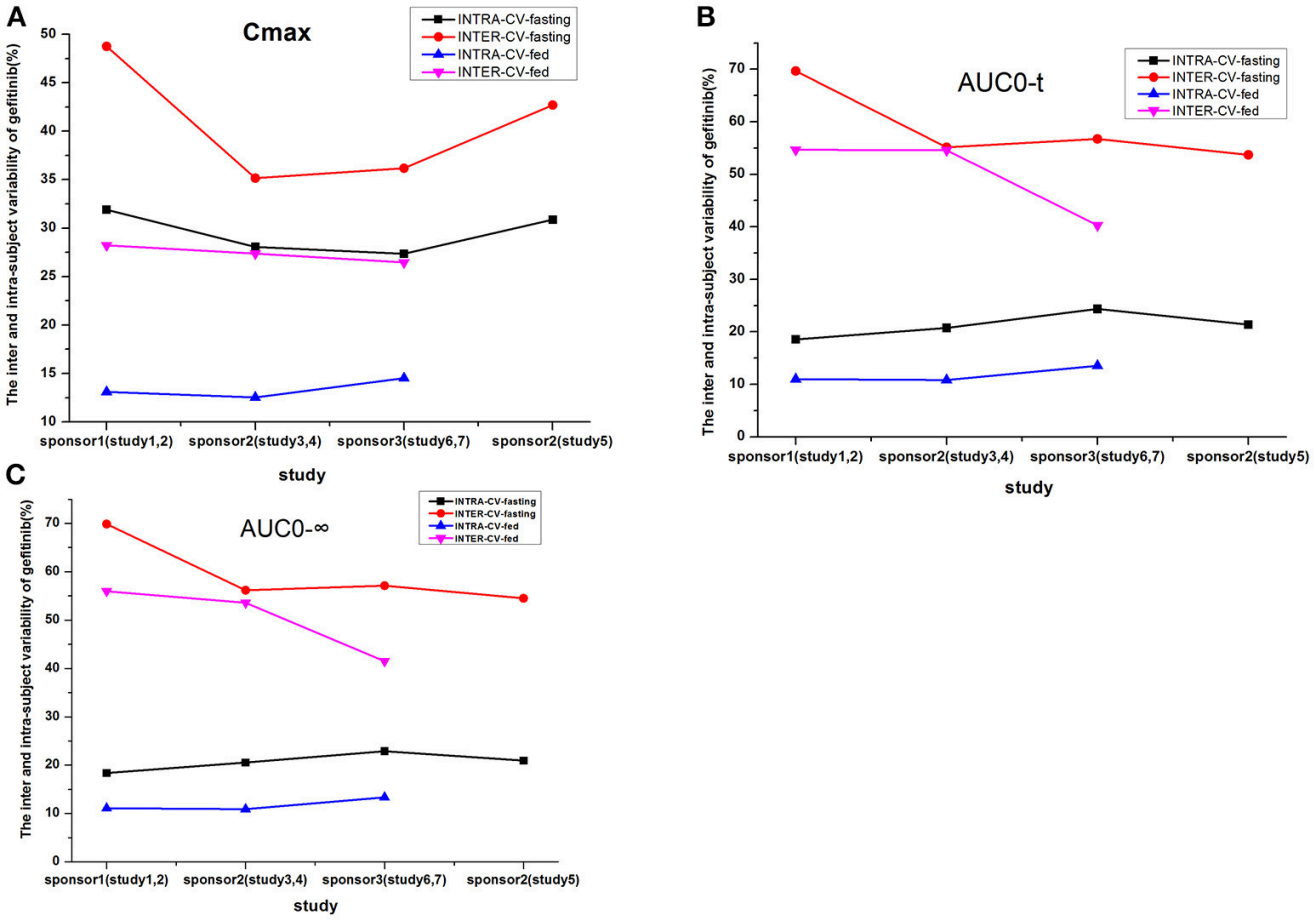
The inter- and intra-subject variability of gefitinib (%) for the following parameters: C_max_ **(A)**, AUC_0−t_ **(B)**, and AUC_0−∞_ **(C)**.

The inter-CV was more than the intra-CV of each pharmacokinetic parameter. The intake of high-fat food could reduce the inter-CV. But the degree of reduction was not significant. The intra-CV varied to a lesser extent compared to the inter-CV, which showed a modestly increased variability in studies 1–7 (Table 5, Figure 4).

**Figure 5:**
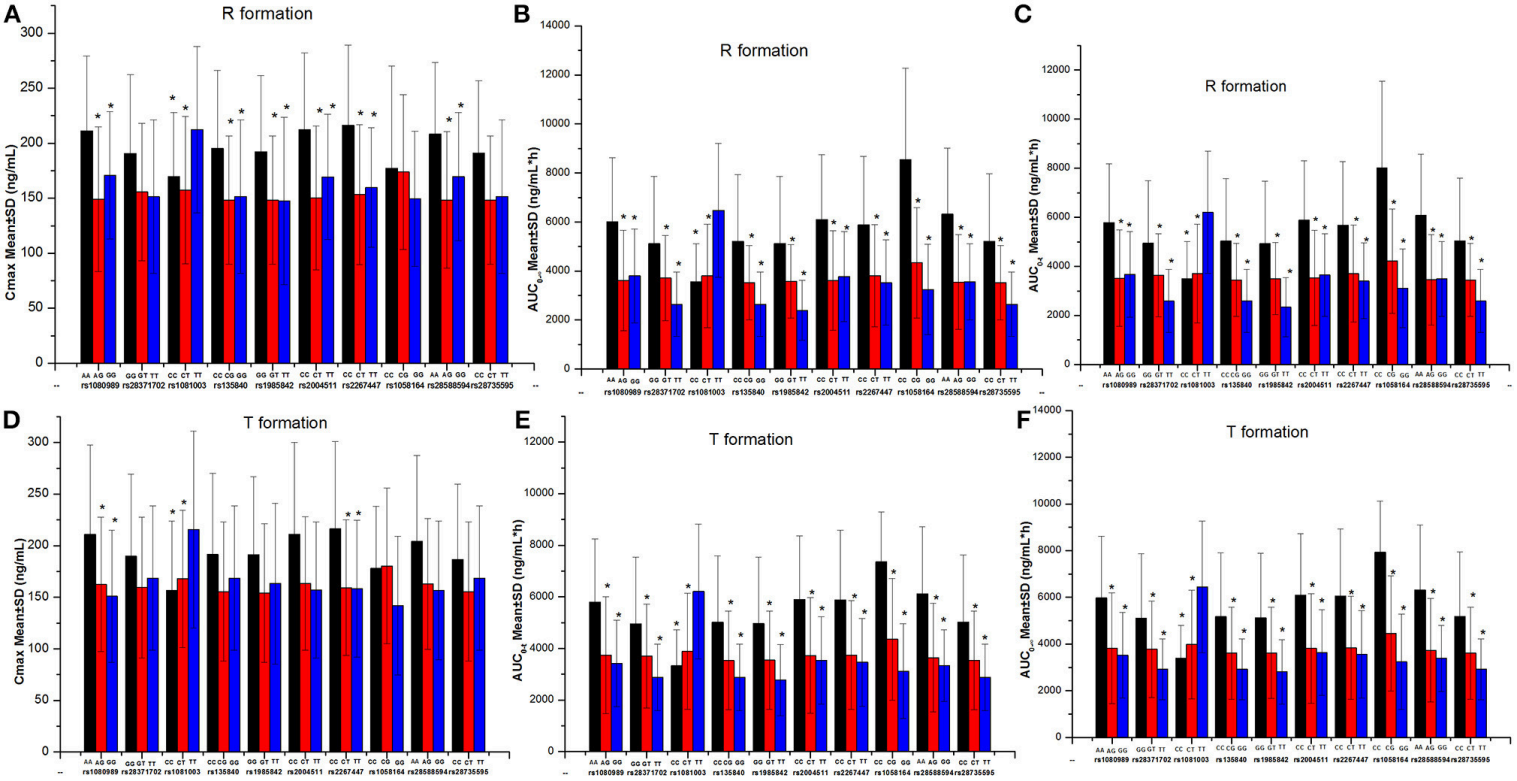
The comparison of gefitinib exposure (C_max_ and AUC) in different genotypes. *P < 0.05 compared with the third genotype (without asterisk) at each single nucleotide polymorphism site. **(A)** The Cmax of R formation. **(B)** The AUC0-∞ of R formation. **(C)** The AUC0-t of R formation. **(D)** The Cmax of T formation. **(E)** The AUC0-t of T formation. **(F)** The AUC0-∞ of T formation.

### Re-estimation of sample size

We re-estimated the sample size of the seven studies based on their bioequivalence results (GMR and intra-CV). The sample size of re-estimation was less than the enrollment size and was about 12 in the fed studies. The sample sizes for the fasting conditions were more complicated. Due to the large intra-CV and the different formulation technologies used by the different sponsors, the sample size of the re-estimation changed too much (Table 5).

### Tolerability and safety analysis

All subjects were able to tolerate the T and R formulations of gefitinib. A total of 69 AEs were reported during the execution of studies 1–7. Forty of these AEs were found to be related to the R product, whereas 29 AEs were related to the T product. It was observed that 44 of these events were related to the studies under fasting conditions and 25 AEs were related to the studies under fed conditions.

There were no specific AEs, and those with the highest frequency were fever and rhinobyon, which were nonspecific symptoms. All AEs were of mild intensity and were mostly related to the administered drug. Seven study subjects received additional medical treatment for fever, urticaria, and abdominal distension. However, the other AEs did not require any additional medical treatment. It is noteworthy that no serious AEs were observed throughout the study. All of the AEs were reported to the institutional Review Board of The First Hospital of Jilin University (Supplementary Tables 1, 2).

## Discussion

Seven studies (1–7) pertaining to gefitinib bioequivalence were completed with drug from three different sponsors. The study designs were randomized, open-label, and 2-period crossover studies in both fasted and fed healthy subjects. Seven studies (1–7) were conducted to investigate the single-dose pharmacokinetic profile of gefitinib tablets in healthy volunteers. It was observed that gefitinib was absorbed slowly, with the peak plasma level occurring at 4–5 h after the dose and coinciding with the T_max_ of cancer patients (3–7 h). The plasma concentrations decreased slowly in a biexponential fashion. The measured t_1/2_ was 18–26 h, which is shorter than that reported previously in cancer patients (41 h)^2^ Moreover, gefitinib showed clearance primarily by the liver, with a total plasma clearance of 595 mL/min after intravenous administration. In studies 1–7, after the oral administration of drug, the clearance was 749–1,286 mL/min. The intravenous pharmacokinetics of gefitinib demonstrated that the drug is extensively distributed into the body tissues and is rapidly cleared from the plasma, with a mean steady state volume of distribution of 1,400 L^3^ In the present studies, it was also noticed that gefitinib was distributed extensively throughout the body with a distribution volume of 1,502–2,219 L. Clearance of a drug by the liver depends on the liver blood flow and the ability of the liver to metabolize the drug, i.e., the hepatic extraction ratio. The liver metabolic abnormalities in cancer patients may be one of the main reasons for a lower clearance of gefitinib; therefore, a higher t_1/2_ is observed in cancer patients than in healthy subjects (Cantarini et al., 2004; Swaisland et al., 2005).

Solubility and permeability are the two main parameters for oral drug absorption; these parameters have been comprehensively studied, and a precise relationship has been well investigated (Davit et al., 2016). The high intestinal permeability manifestation of gefitinib and the lower solubility (dissolution rate) in the intestines make gefitinib a Biopharmaceutics Classification System (BCS) class II compound (Davit et al., 2016). Gefitinib undergoes rapid dissolution in acidic media, but the solubility decreases with an increase of pH, until the pH is neutral in the intestines. The reported oral bioavailability of gefitinib in healthy male volunteers is 57%, but manipulating the gastric pH up to 5 resulted in a 47% reduction of the relative bioavailability (Swaisland et al., 2005; Davit et al., 2016). In the current investigation, our results convincingly revealed that the exposure was higher in the studies under fed conditions compared to the studies conducted in the fasting state. Moreover, the fed conditions could alter the gastric pH, which could increase the dissolution and absorption by up to 12–58%.

The intra-subject variability in healthy male subjects was 30% and 12–14% under fasting and fed conditions, respectively. Thus, the intra-subject variability in the current study was in close agreement with previous reports (Bergman et al., 2007). It can be inferred that the pharmacokinetics of gefitinib are highly variable under fasting conditions because an intra-CV greater than 30% during the investigation of bioavailability parameters was found (Karalis et al., 2012). This high variability can be ascribed either to the drug substance itself or it can be secondary to the drug product formulation. The underlying causes of high variability include physiological and pathological conditions as well as physicochemical properties of the drug product. The physiological factors are related to pH, pancreatic or bile secretions, gastric emptying, intestinal motility, luminal/mucosal enzymes, and circadian rhythm; all of these factors can vary significantly between different subjects but also within the same subjects. Other factors that can influence absorption are age, gender, drug interactions, and food intake (Karalis et al., 2008, 2010). In the current study, in addition to the same batch of drug being administered in each study from the same sponsor, the study subjects were all male, healthy adults with a similar age, BMI, and weight. The intra-CV may arise from a gastrointestinal factor or the physicochemical properties of gefitinib. However, high-fat food intake reduced the intra-CV significantly, indicating that gastrointestinal factors are the primary reason for the highly variability.

The observed inter-CV was very large in the present studies, regardless of the food intake before dosing. The difference of the inter-individual pharmacokinetic profiles might be explained by the inter-CV in gastric emptying and/or precipitation/dissolution of gefitinib in the proximal small intestine, low jejunal pH, increased expression of enzymatic and transporter activity, or rapid small intestinal transit. However, no pronounced difference was observed in gastric emptying, precipitation, or re-dissolution of gefitinib in the proximal human jejunum between the high- and low-exposure subjects (Bergman et al., 2007). The low jejunal pH and rapid small intestinal transit were not possible because both of the fasting and fed studies exhibited a high inter-CV. Furthermore, gefitinib undergoes extensive hepatic metabolism in humans, predominantly by CYP3A4, which metabolizes the *N*-propoxymorpholino group. At the same time, the generation of *O*-desmethyl gefitinib is the major active component produced by CYP2D6, which accounts for 14% of the dose (Li et al., 2006; Arafa and Atteia, 2018; Kim et al., 2018)^1^.

Polymorphisms in various metabolic enzymes and/or transport proteins may contribute to the high inter-CV (Cantarini et al., 2008)^4^ We found that CYP2D6 gene polymorphisms in studies 3 and 5 could be associated with the exposure, and a major mutation occurred in the intron variant region. Additionally, different activities of cytochrome enzymes could contribute to different exposures and a higher inter-CV. A 39% decrease in the gefitinib AUC in CYP2D6 ultra rapid metabolizers vs. extensive metabolizers has been reported (Chen et al., 2018). However, the type of metabolizer did not influence the bioequivalence results due to the higher inter-CV observed in study 5. The inter-CV of study 5 was higher than that of study 3; thus, study 5 achieved bioequivalence (Swaisland et al., 2006; Karalis et al., 2008; Davit et al., 2012).

The products or formulations are considered bioequivalent if the difference between the two parameters being compared is statistically insignificant (*P* ≥ 0.05) and the 90% CI for these parameters fall within 80–125%. According to the guidelines, the 90% CI of the GMR (AUC and C_max_) must fall within the limits of 80–125%. These limits are prescribed by the following equation:

(1) $$\begin{array}{l} 90\%CI\;=EXP\left(Diff\pm 0.05t,N-2SQRT\left(\sigma i^{2}\times 2/N\right)\right) \end{array}$$

Where Diff represents the difference between the T and R means of the logarithmically transformed metric μT and μR, σi^2^ is the residual (within-subject) variance of the logarithmically transformed characteristics (calculated as the mean square error of ANOVA), and N is the number of participants in the bioequivalence study. In any case, the CI is directly proportional to the intra-CV and inversely proportional to the number of participants (*N*). Therefore, in study 5, we enlarged the Diff and sample size (*N*) without changing the drug batch or production process (Haidar et al., 2008; Ramirez et al., 2008; Tothfalusi et al., 2009; Baek et al., 2010; Tothfalusi and Endrenyi, 2016, 2017). Then, we were able to get the bioequivalence result between the T and R formulations.

Finally, we re-estimated the sample size for each study and achieved bioequivalence. We found that the fed study did not need as large of a sample size and that the fed condition could reduce the intra-CV. In the future, we suggest that 18 subjects may be sufficient for the fed study, considering the intra-CV (12–14%) (Bergman et al., 2007; Wilson et al., 2009).

In general, AEs are related to the dose and exposure (Hirose et al., 2016), so in individuals without active CYP2D6, the high exposure could contribute to AEs. We also analyzed the exposure of gefitinib (C_max_ and AUC) in the subjects with AEs and without AEs in each study. However, no difference (data not shown) was noticed. The AEs were all nonspecific in present study, indicating that gefitinib was well tolerated (Gross et al., 2012). These bioequivalence results for gefitinib under fasting and fed conditions suggest that the T formulation could give similar exposure to that of the R formulation, with good reproducibility in individual patients.

## Conclusions

The data obtained from the seven studies (1–7) demonstrated that the highly variable drug gefitinib is well tolerated in healthy male subjects. The results exhibited that gefitinib (250-mg tablet) is orally bioavailable in healthy study subjects under fasting and fed conditions. Gefitinib bioequivalence was easier to achieve under fed conditions than under fasting conditions. We further suggest that when the bioinequivalence is due to the 90% CI of the GMR being outside of the limits, the sample size can be enlarged without changing the dosage formulation to narrow down the range to achieve bioequivalence.

## Author contributions

XL, SH, HZ, and YD designed the experiment. XL, SH, HZ, MW, XZ, and CJL performed the clinic trials. HZ and CYL analyze the data. QL, ZS, HZ, and YD write and edit the paper, and draw the figures.

### Conflict of interest statement

The authors declare that the research was conducted in the absence of any commercial or financial relationships that could be construed as a potential conflict of interest.

## Footnotes

^1^https://www.fda.gov/downloads/drugs/guidancecomplianceregulatoryinformation/guidances/ucm495026.pdf.

^2^https://www.accessdata.fda.gov/drugsatfda_docs/label/2005/021399s008lbl.pdf.

^3^https://www.accessdata.fda.gov/drugsatfda_docs/label/2005/021399s008lbl.pdf.

^4^https://www.accessdata.fda.gov/drugsatfda_docs/label/2005/021399s008lbl.pdf.
