# Supplementary material for: Association of Variability and Pharmacogenomics With Bioequivalence of Gefitinib in Healthy Male Subjects
Source: Front Pharmacol. 2018 Aug 7;9:849. doi: 10.3389/fphar.2018.00849 (PMC6090208; doi:10.3389/fphar.2018.00849)
Supplement: Supplementary file 1 [file Table_1.docx]

**Supplementary Table 1**. Incidence of adverse events

| **Adverse events** | **Reported**  **incidence (fed)** | | **Reported**  **incidence (fasting)** | |
| --- | --- | --- | --- | --- |
|  | **Test** | **Reference** | **Test** | **Reference** |
| Alanine aminotransferase increased | 0 | 1 | 1 | 1 |
| Gamma-glutamyl transpeptidase increased | 1 | 0 | 0 | 0 |
| Aspartate aminotransferase increased | 2 | 0 | 0 | 1 |
| Creatine kinase increased | 0 | 1 | 1 | 1 |
| Hyperkalemia | 0 | 2 | 0 | 2 |
| Fasting blood-glucose increased | 2 | 1 | 0 | 1 |
| Lactic dehydrogenase increased | 0 | 0 | 0 | 1 |
| Total bile acid increased | 0 | 0 | 0 | 2 |
| The white blood cell count decreased | 0 | 1 | 1 | 0 |
| The white blood cell count increased | 1 | 0 | 2 | 2 |
| Erythropoiesis decreased | 1 | 1 | 0 | 1 |
| The absolute value of neutrophils decreased | 0 | 1 | 1 | 1 |
| The absolute value of neutrophils increased | 1 | 0 | 2 | 2 |
| The absolute value of lymphocyte decreased | 0 | 1 | 0 | 0 |
| Diarrhea | 1 | 1 | 0 | 1 |
| Rash | 1 | 1 | 0 | 1 |
| Ventricular extrasystole | 0 | 1 | 1 | 0 |
| Vomit | 0 | 1 | 0 | 0 |
| Bradycardia | 0 | 1 | 0 | 0 |
| Middle finger crush injury | 1 | 0 | 0 | 0 |
| Maculopapule | 0 | 0 | 0 | 1 |
| Rhinobyon | 0 | 0 | 2 | 1 |
| Albuminuria | 0 | 0 | 2 | 1 |
| Fever | 0 | 0 | 5 | 3 |
| Abdominal distension | 0 | 0 | 0 | 2 |
| Hypertension | 0 | 0 | 0 | 1 |

**Supplementary Table 2** The distribution of CYP2D6 genotypes

| **AssociatedGene** | **Functionof the gene** | **SNP** | [Allele Origin](http://www.ncbi.nlm.nih.gov/projects/snp/docs/rs_attributes.html#alleleorigin) | **Mutation rate*** | **Functional Consequence** | **Genotype1** | **n1** | **Genotype 2** | **n2** | **Genotype 3** | **n3** |
| --- | --- | --- | --- | --- | --- | --- | --- | --- | --- | --- | --- |
| CYP2D6 | Metabolism | rs1080989 | G | 39% | Intron variant | AA | 22 | AG | 44 | GG | 22 |
| CYP2D6 | Metabolism | rs28371702 | G | 34% | Intron variant | GG | 37 | GT | 43 | TT | 9 |
| CYP2D6 | Metabolism | rs1081003 | C | 42% | Intron variant | CC | 23 | CT | 51 | TT | 15 |
| CYP2D6 | Metabolism | rs1985842 | G | 43% | Intron variant | GG | 44 | GT | 39 | TT | 7 |
| CYP2D6 | Metabolism | rs2004511 | T | 58% | Intron variant | CC | 21 | CT | 45 | TT | 24 |
| CYP2D6 | Metabolism | rs2267447 | T | 43% | Intron variant | CC | 21 | CT | 49 | TT | 19 |
| CYP2D6 | Metabolism | rs1058164 | G | 56% | Intron variant  synonymous Codon | CC | 3 | CG | 68 | GG | 16 |
| CYP2D6 | Metabolism | rs28588594 | G | 41% | Intron variant  synonymous codon | AA | 20 | AG | 45 | GG | 23 |
| CYP2D6 | Metabolism | rs28735595 | T | 56% | Intron variant | CC | 39 | CT | 40 | TT | 9 |
| CYP2D6 | Metabolism | rs135840 | NA | NA | Intron variant | CC | 40 | CG | 40 | GG | 9 |

*CYP2D6: Cytochrome P450 2D6. The mutation rate of each SNP comes from [*http://www.ncbi.nlm.nih.gov/snp/*](http://www.ncbi.nlm.nih.gov/snp/)
